# Extracellular Vesicles in Gut‐Bone Axis: Novel Insights and Therapeutic Opportunities for Osteoporosis

**DOI:** 10.1002/smsc.202400474

**Published:** 2024-12-23

**Authors:** Han Liu, Ruiyang Li, Huijian Yang, Bo Situ, Guangchao Wang, Ke Xu, Jiacan Su

**Affiliations:** ^1^ Department of Orthopedics, Xinhua Hospital Shanghai Jiao Tong University School of Medicine Shanghai 200092 China; ^2^ Institute of Translational Medicine Shanghai University Shanghai 200444 China; ^3^ Organoid Research Center Shanghai University Shanghai 200444 China; ^4^ National Center for Translational Medicine (Shanghai) SHU Branch Shanghai University Shanghai 200444 China; ^5^ Department of Clinical Laboratory Shanghai Zhongye Hospital Shanghai 200941 China; ^6^ Department of Laboratory Medicine Nanfang Hospital Southern Medical University Guangzhou 510515 China

**Keywords:** bacterial extracellular vesicles, gut microbiota, intestinal organoids, organoids extracellular vesicles, osteoporosis

## Abstract

Osteoporosis (OP) is a systemic and retrogressive disease characterized by decreased bone density and fragile bone microstructure. Extracellular vesicles (EVs), a cell‐free system with a phospholipid bilayer released by cells that cannot be replicated, have unique nanostructure, stable drug‐loading capacity, and good biocompatibility, playing an important role in regulating the gut‐skeletal axis. A growing body of research demonstrates that gut microbiota (GM) influence the development of OS, while bacteria‐derived EVs (BEVs) have become a new dialogue medium between the gut and bone. Additionally, organoids are 3D cell clusters in vitro that highly simulate the structure and function of corresponding organs. Intestinal organoids‐derived EVs (IOEVs) serve as another promising communication medium between the gut and bone due to their significant physiological effects. Herein, three types of gut‐bone axes, including the traditional, BEVs‐based, and IOEVs‐based gut‐bone axes are innovatively proposed. The impact of the BEVs‐based and IOEVs‐based gut‐bone axes on OP is focused. The comprehensive summary of three types of gut‐bone axes will reveal the relationship between intestinal and bone and provide new solution to OP therapy.

## Graphical abstract

Extracellular vesicles (EVs) are cell‐released nanocarriers with a phospholipid bilayer structure that play key roles in regulating physiological and pathological processes. Both intestinal microorganisms and intestinal organoids can secrete EVs, namely, bacterial extracellular vesicles (BEVs) and intestinal organoid extracellular vesicles (IOEVs). Both natural and engineered BEVs or IOEVs can regulate the development of osteoporosis and the bone metabolism.

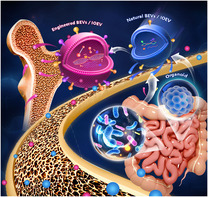

## 1 Introduction

With the rapid development of population aging, osteoporosis (OP) and osteoporosis fracture (OPF) are increasing, seriously affecting the quality of life of the elderly.^[^ ^1^ ^]^ OP is a retrogressive disease characterized by decreased bone density and fragile bone microstructure that is prone to OPF.^[^ ^2^ ^]^ OP has a high disability rate and is combined with multiple diseases, which have gradually become the most important causes of disability and death in the elderly.^[^ ^3^ ^]^ OP frequently arises from an imbalance between the formation of bone by osteoblasts and the resorption of bone by osteoclasts.^[^ ^4^ ^]^ Currently, drugs used for the treatment of OP exhibit low bioavailability and long‐term toxicity, thereby impeding their broader application.^[^ ^5^ ^]^ Consequently, there exists an urgent necessity to devise a novel and efficacious therapeutic strategy for addressing OP.

Numerous studies have shown that gut microbiota (GM) can modulate the bone metabolism directly and indirectly through multiple pathways, including the release of metabolites and interactions with immune cells or hormones.^[^ ^6^ ^]^ Recently, GM‐derived extracellular vesicles (EVs) have been recognized as factors directly affecting bone metabolism.^[^ ^7^ ^]^ EVs, nonreplicating lipid bilayer particles released from cells, contain cell‐specific proteins, lipids, and nucleic acids, which can be transferred as signaling molecules to alter the functions of recipient cells.^[^ ^8^ ^]^ Recent research has highlighted the crucial roles of EVs in various physiological and pathological processes, such as antigen presentation in immunity, tumor growth and metastasis, and tissue repair.^[^ ^9^ ^]^ Different types of EVs secreted by various cells have distinct compositions and functions, serving as potential biomarkers for disease diagnosis.^[^ ^10^ ^]^ Therefore, EVs have become a new generation of drug delivery carriers and communication media due to their special nanostructure, stable drug loading, and good biocompatibility,^[^ ^11^ ^]^ which regulate bone metabolism by delivering endogenous cargo, such as nucleic acids and proteins, into host cells.^[^ ^12^ ^]^ The emerging bacterial‐derived EVs (BEVs)‐based gut‐bone axis has provided new solutions for the treatment of OP.^[^ ^13^ ^]^ However, both bacterial derivatives‐based gut‐bone axis (directly) and immune and endocrine system‐based gut‐bone axis (indirectly) may not fully reveal the relationship between gut and bone.

Organoids are three‐dimensional (3D) cell clusters in vitro that highly simulate the structure and function of corresponding organs in the body^[^ ^14^ ^]^ and have been widely used in precision medicine, regenerative medicine, disease modeling, and host–microbe interaction.^[^ ^15^ ^]^ Intestinal organoids, as the first organoids to be constructed,^[^ ^16^ ^]^ have been considered as new therapeutic models for complex diseases.^[^ ^17^ ^]^ With the rapid development of organoid technology, intestinal organoids have been able to highly simulate intestinal tissue.^[^ ^18^ ^]^ Organoids EVs (OEVs) have also become a novel and promising tool for investigating the communication between cells.^[^ ^19^ ^]^ OEVs, including intestinal OEVs (IOEVs), are promising communication media and nanocarriers due to their significant biological functions and physiological effects, great biocompatibility, and stable loading capacity.^[^ ^20^ ^]^ Therefore, IOEVs may provide a completely different idea for the gut‐bone dialogue mechanism. IOEVs‐based gut‐bone axis may also provide an innovative and comprehensive solution for the treatment of OP. Importantly, in addition to the abovementioned gut‐bone axis, the incorporation of IOEVs‐based gut‐bone axis has the potential to elucidate the relationship between gut and bone in a more comprehensive and systematic manner.

In this review, we systematically summarized the gut‐bone axis from three levels, including traditional metabolites and immunoendocrine regulation, BEVs, and IOEVs. The traditional gut‐bone axis mainly utilizes GM‐derived metabolites and immune and endocrine systems to regulate bone metabolism. Importantly, BEVs‐based and IOEVs‐based gut‐bone axes provide another strategy for OP therapy. This innovative segmentation perspective helps to understand the gut‐bone axis systematically and comprehensively. Three types of gut‐bone axes models will comprehensively reveal the relationship between gut and bone and provide complete insights into OP therapy.

## 2 Traditional Gut‐Bone Axis for OP Therapy

Bone is a dynamic tissue that sustains normal bone mass and density by regulating the balance between bone formation facilitated by osteoblasts and bone resorption orchestrated by osteoclasts, which is the key to maintaining normal bone mass and density.^[^ ^21^ ^]^ Recently, GM has been proven to be an important regulator of bone formation and resorption balance.^[^ ^22^ ^]^ GM, mainly colonized in the host intestine, participates in maintaining the host homeostasis by regulating various physiological processes, such as intestinal development, nutrient digestion, immune regulation, and endocrine regulation.^[^ ^23^ ^]^ Many studies have shown that GM can affect bone metabolism and the progression of OP.[^6^, ^22^, ^24^] Given the importance of GM in regulating bone health, it is of great significance to critically summarize the communication mechanisms between GM and bone. Here, we provide a comprehensive summary of the traditional gut‐bone axis, where GM affects bone metabolism directly through metabolites and indirectly through immune and endocrine systems.

### 2.1 Metabolites‐Based Gut‐Bone Axis for OP Therapy

**Figure 1:**
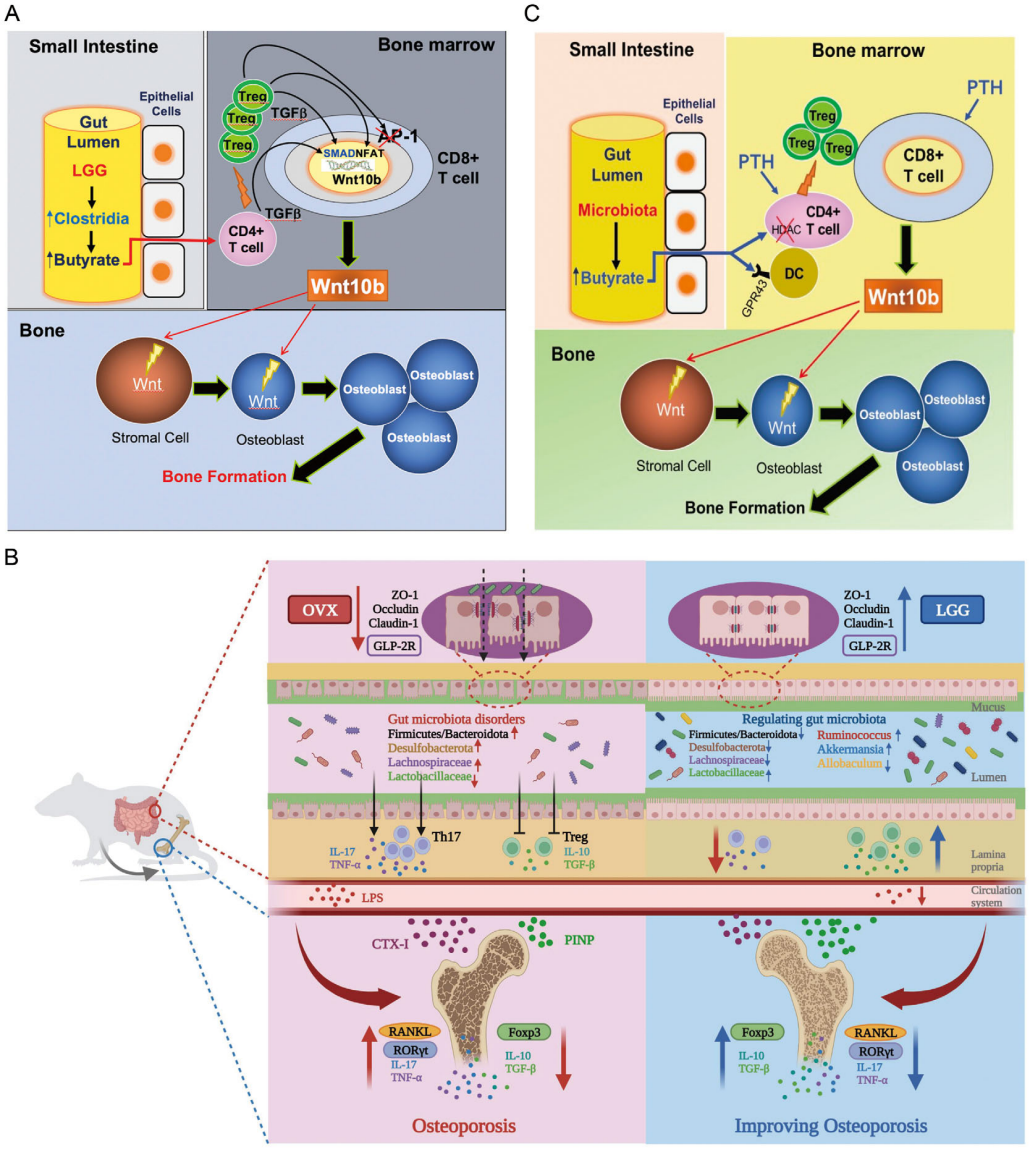
GM affects bone metabolism directly through metabolites and indirectly through immune and endocrine systems. A) Schematic diagram of LGG supplementation regulating OP through pyruvate. Reproduced with permission.^[28b]^ Copyright 2018, Cell Press. B) Schematic diagram of LGG supplementation regulating OP through immune systems. Reproduced with permission.^[6a]^ Copyright 2023, Taylor & Francis Group, LLC. C) Schematic diagram of GM regulating OP through endocrine systems. Reproduced with permission.^[^ ^40^ ^]^ Copyright 2020, American Society for Clinical Investigation.

GM, resided in the luminal stream or gut mucosa, can afford the energy derived from foodstuff, regulate epithelial growth, eliminate pathogens colonization, and promote the maturation of immune systems.^[^ ^25^ ^]^ GM also influences the gut‐bone axis, with studies showing that sex steroid deficiency‐induced bone loss can be prevented by probiotics.[^24^, ^26^] GM affects bone metabolism by producing metabolites that enter the bloodstream, with short‐chain fatty acids (SCFAs) being key regulators.^[^ ^27^ ^]^ SCFAs like propionate and butyrate influence osteoclast metabolism, calcium absorption, and mineral solubility, ultimately improving bone health.^[^ ^28^ ^]^ Tyagi et al.^[28b]^ found that the supplementation of *Lactobacillus rhamnosus* GG (LGG) increased the content of metabolite butyrate, which improved OP via T regulatory (Treg) cell‐mediated regulation of Wnt10B signaling pathway (**Figure** **1**A). Additionally, insulin‐like growth factor 1 (IGF‐1),^[^ ^29^ ^]^ hydrogen sulfide (H_2_S),^[^ ^30^ ^]^ vitamins,^[^ ^31^ ^]^ and polyamines^[^ ^32^ ^]^ are reported to regulate bone metabolism through various mechanisms, such as stimulating bone formation, preserving osteogenic differentiation of cells, enhancing calcium metabolism, and promoting bone volume. These findings highlight the important connection between GM and bone health.

Although these bacterial metabolites participated in the process of OP through different mechanisms, regulating the balance of osteoblasts and osteoclasts was still the fundamental basis for preventing and treating OP.^[^ ^33^ ^]^ Taken together, the metabolite‐based gut‐bone axis provided a potential therapeutic option for OP treatment (**Table** **1** **).**

**Table 1:** Recent metabolites‐based, BEVs‐based, and IOEVs‐based gut‐bone axes for OP therapy.

Different gut‐bone axis	Contributions	References
Metabolites‐based		
Propionate	Inducing metabolic reprogramming of osteoclasts	[28a]
H_2_S	Reducing oxidative stress and stimulating the osteogenic differentiation of stromal cells	[100]
Butyrate	Inhibiting histone deacetylases (HDACs) and reducing osteoclastogenesis	[101]
Butyrate	Stimulating mineralized nodule formation and osteoprotegerin expression	[102]
Butyrate	Stimulating bone formation via Treg cell‐mediated regulation of Wnt10b pathway	[28b]
Lipopolysaccharide	Inhibiting osteoblast differentiation by suppressing the expression of transcriptional factors, Runt‐related transcription factor 2(RUNX2), Sp7 transcription factor (Sp7, Osterix), and activating transcription factor 4 (ATF4)	[103]
		[104]
Acetate	Enhancing bone formation by stimulating early osteoblastic differentiation in bone	
Polyamines	Regulating gene expression of Runx2 and osteopontin	[105]
Polyamines	Modulating nitric oxide production and COX‐2 gene expression and enhancing osteogenic differentiation of stem cells	[106]
Polyamines spermidine	Disrupting the differentiation and maturation of osteoclasts	[107]
Sodium hydrosulfide	Inhibiting osteoclast progenitor cells differentiation via NRF2‐dependent mechanism	[108]
Lithocholic acid	Reducing osteoblast viability via vitamin D	[109]
BEVs‐based		
miRNA (natural BEVs)	Enhancing osteogenic activity and inhibiting osteoclasts formation	[22a]
miRNA (natural BEVs)	Inhibiting osteoclasts formation	[53]
miRNA (engineered BEVs)	Delivering endogenous miRNA to bone by bone‐targeting peptides	[59]
siRNA (engineered BEVs)	Delivering SOST siRNA to bone by CXCR4	[58]
BMP‐2 (engineered BEVs)	Targeted delivery of recombinant BMP‐2 by CXCR4	[58]
IOEVs‐based		
miRNA (natural BEVs)	Regulating inflammatory responses of immune cells in vitro	[78]
miRNA (natural BEVs)	Improving IBD symptoms and improved bone mass	This review
miRNA/siRNA/drug	Delivering miRNA/siRNA/drug by engineering method	This review

### 2.2 Immune‐Based and Endocrine‐Based Gut‐Bone Axis for OP Therapy

In addition to direct metabolites‐based gut‐bone axis, GM can also influence bone metabolism indirectly through both immune and endocrine systems‐based gut‐bone axes. Osteoimmunology underscores the mechanisms and intricate communication between the immune and skeletal systems.^[^ ^34^ ^]^ The immune‐based gut‐bone axis highlights the impact of GM on the balance between T helper 17 (Th17) and T regulatory (Treg) cells, where the modulation of this balance can affect osteoclast‐mediated bone resorption and osteoblast‐mediated bone formation.^[^ ^35^ ^]^ Studies have shown that GM plays a crucial role in regulating Th17/Treg balance and can be targeted for OP therapy.^[^ ^36^ ^]^ Recently, Guo et al.^[6a]^ found that LGG improves estrogen deficiency‐induced OP by regulating the GM and intestinal barrier and Th17/Treg balance in the intestine and bone (Figure 1B). In summary, understanding the immune‐based gut‐bone axis and revealing the role of Th17/Treg balance will provide new insights for OP therapy.

In contrast, the endocrine‐based gut‐bone axis emphasizes the role of GM in controlling sex steroid levels and impacting estrogen‐dependent bone metabolism. GM was also considered as an “endocrine organ”.^[^ ^37^ ^]^ Wang et al.^[^ ^38^ ^]^ found that *Prevotella histicola* could improve estrogen deficiency‐induced bone loss by modulating gut permeability and inhibiting osteoclast activity. Ridlon et al.^[^ ^37^ ^]^ even proposed the “sterolbiome” to represent the potential of GM to produce endocrine molecules. In general, estrogen regulates bone metabolism by downregulating the GM, GM‐driven immune response, and balancing osteoblasts and osteoclasts. Moreover, GM also interacts with hormones like parathyroid hormone (PTH), influencing calcium balance and bone remodeling.^[^ ^39^ ^]^ Li et al.^[^ ^40^ ^]^ demonstrated that the microbiota, especially the levels of butyrate generated by GM, was required for intermittent PTH to stimulate bone formation and increase bone mass (Figure 1C). In summary, understanding the immune‐based and endocrine‐based gut‐bone axes will provide new insights for OP therapy.

## 3 BEVs‐Based Gut‐Bone Axis for OP Therapy

**Figure 2:**
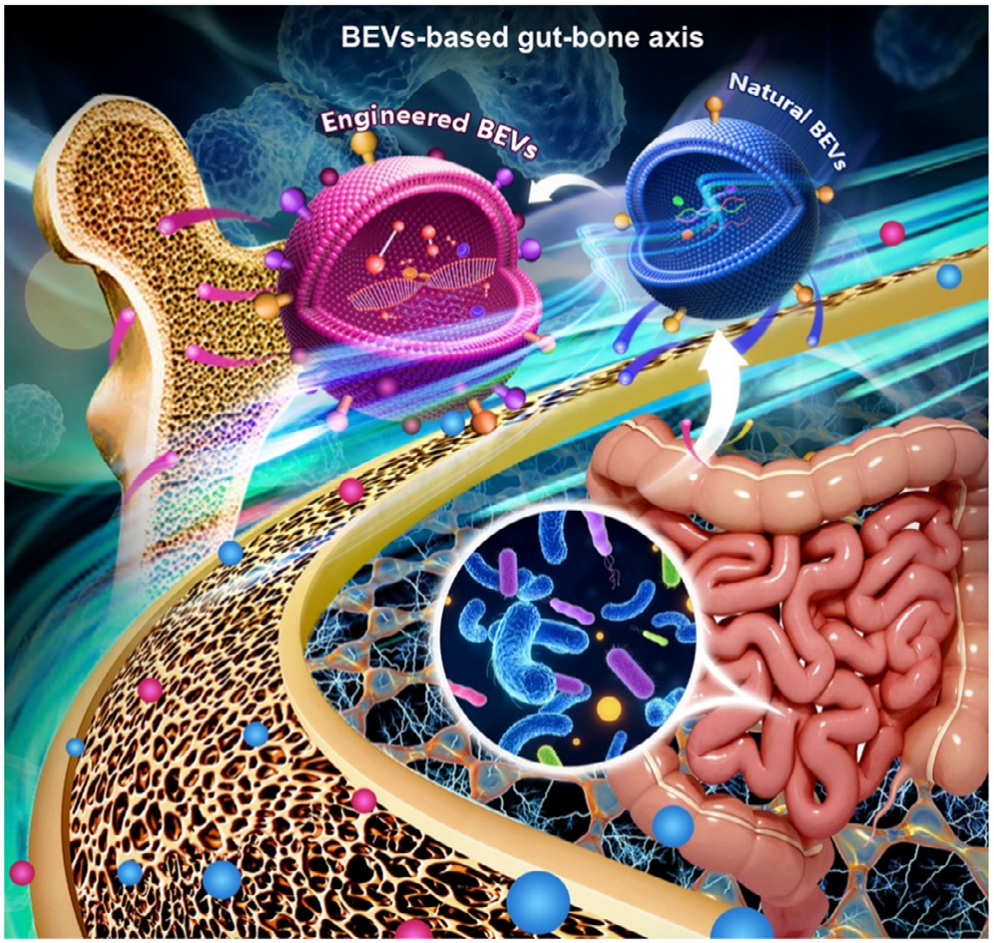
Schematic illustration of BEVs‐based gut‐bone axis. GM affects bone metabolism through BEVs. The blue nanoparticle represents natural BEVs, while the red nanoparticle represents engineered BEVs. Both natural and engineered BEVs can regulate the development of OP and the bone metabolism.

BEVs, nanocarriers with a phospholipid bilayer secreted by bacteria, regulate communication between bacteria and cells by delivering endogenous bioactive agents, such as nucleic acids, proteins, and lipids.[^7^, ^41^] The BEVs‐based gut‐bone axis has received widespread attentions due to their special nanostructure, cell‐free system, impressive great drug loading capacity, minimal toxicity, and excellent biocompatibility.^[^ ^42^ ^]^ Notably, synthetic biology has endowed BEVs with customizability and scalability, making BEVs a strong candidate for clinical application in orthopedics. BEVs‐based gut‐bone axis provides a new insight into the treatment of OP (**Figure** **2**). Therefore, we first provide a comprehensive summary of BEVs. Then we focused on the application of natural and engineered BEVs‐based gut‐bone axis in the treatment of OP.

### 3.1 Overview of BEVs

#### 3.1.1 The Biogenesis, Types, and Composition of BEVs

**Figure 3:**
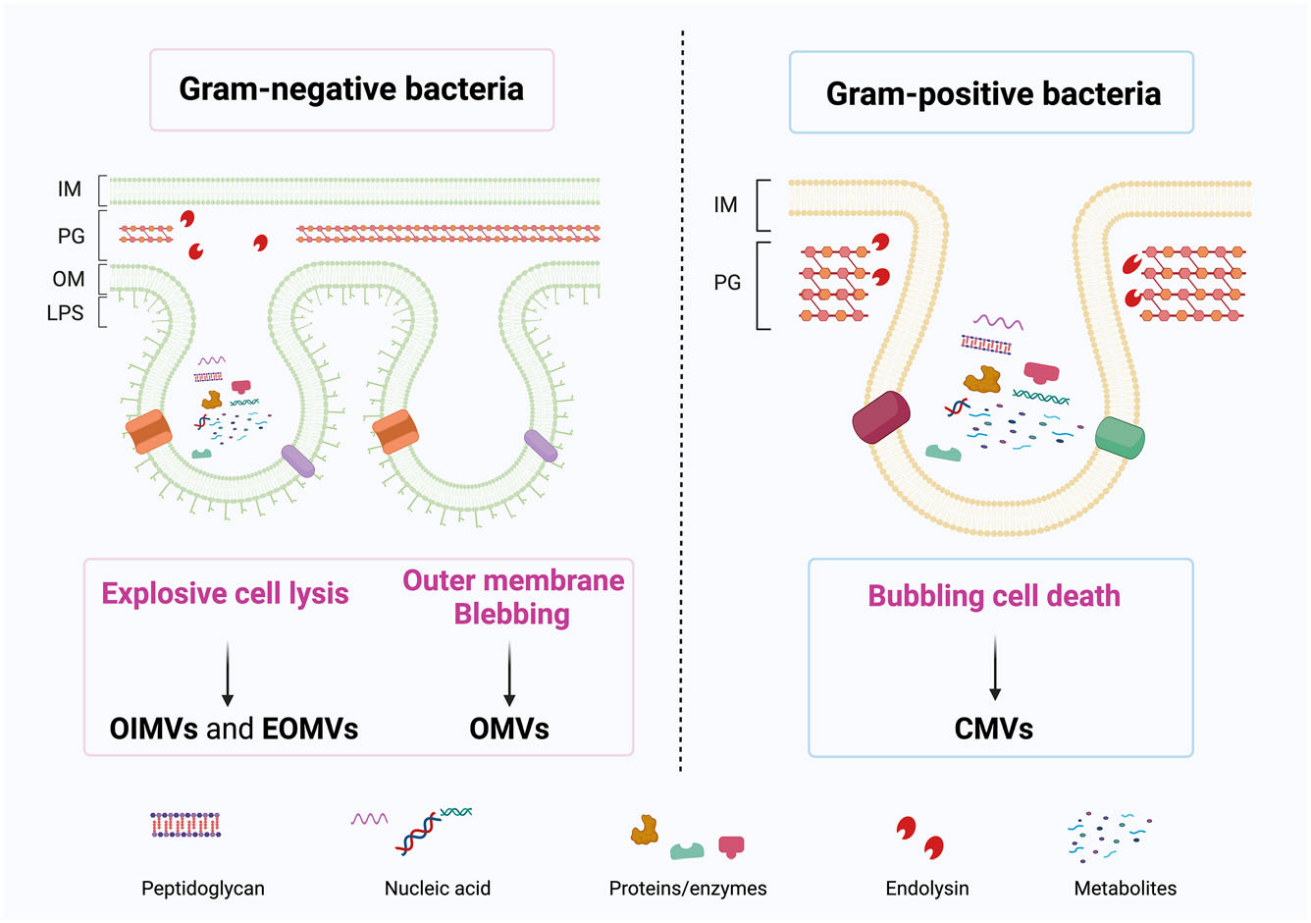
The biogenies, classes, and compositions of BEVs. Gram‐negative bacteria produce OMVs through outer membrane blebbing and generate OIMVs and EOMVs through explosive cell lysis. Gram‐positive bacteria produce CMVs through a mechanism of bubbling cell death. Figure was created with https://app.biorender.com/.

For a better understanding of the application and potential of BEVs in OP, we comprehensively reviewed the biogenesis, types, and composition of BEVs. Beneficial, harmful, and neutral bacteria derived from the GM can be classified as either Gram‐positive or Gram‐negative based on their morphology, structure, and staining characteristics. BEVs were first discovered in Gram‐negative bacteria in the 1960s, however, BEVs were not reported in Gram‐positive bacteria until thirty years later.^[^ ^43^ ^]^ Initially perceived as mere waste disposal stations, BEVs are now understood as communication mediators capable of transporting cargo to other cells and impacting tissue functions across multiple dimensions.^[^ ^44^ ^]^ Both Gram‐positive and Gram‐negative bacteria are capable of producing spherical nanoparticles ranging from 20 to 400 microns in diameter.^[^ ^45^ ^]^ Gram‐positive bacteria produce BEVs known as cytoplasmic membrane vesicles (CMVs) through a mechanism of bubbling cell death,^[^ ^46^ ^]^ whereas Gram‐negative bacteria produce various types of BEVs, including outer membrane vesicles (OMVs) through outer membrane blebbing, and both outer‐inner membrane vesicles (OIMVs) and explosive outer‐membrane vesicles (EOMVs) through explosive cell lysis^[^ ^47^ ^]^ (**Figure** **3**). The composition of BEVs varies depending on their mechanism of formation. Generally, the biggest difference in contents between Gram‐positive and Gram‐negative bacteria lies in the presence of lipo poly saccharide (LPS). Moreover, OMVs predominantly contain outer membrane proteins and LPS, while CMVs and other types of BEVs are enriched in peptidoglycan, nucleic acids, and proteins (Figure 3). It is worth noting that the BEVs produced by probiotics *Escherichia coli* Nissle 1917, a special Gram‐negative bacterium that is often used as chassis cells, do not contain intact LPS.^[^ ^48^ ^]^ Additionally, by knocking out genes such as *msbA*, *msbB*, *lpxL1*, or *lpxM* in most Gram‐negative bacteria, or by utilizing Gram‐positive bacteria to produce BEVs, it is possible to avoid the adverse reactions caused by LPS, thereby enhancing their suitability for disease treatment.[^13^, ^49^] There have been numerous studies using attenuated strains or probiotic derived BEVs for bone disease.[^12^, ^22^]

#### 3.1.2 The Isolation of BEVs

**Figure 4:**
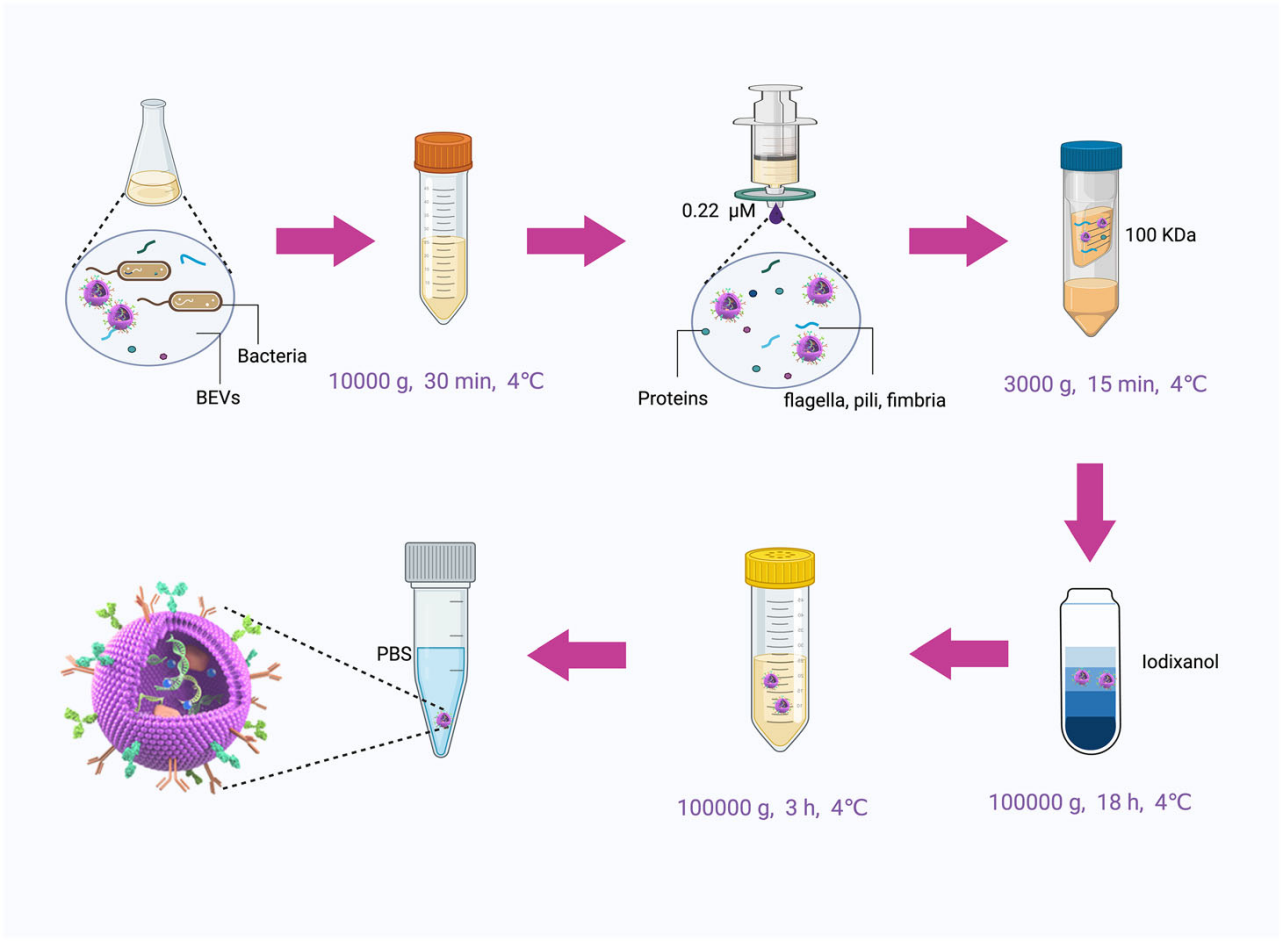
The isolation of BEVs. Bacteria in the culture medium were first removed by low‐speed centrifugation (10 000 g, 30 min) and then sterile filtered using a 0.22 μm filter. The supernatant is then transferred to an UF membrane (100 KDa) and further concentrated via centrifugation at 3000 g for 15 min. For better isolation of BEVs, a combination of UC and DGC was used. BEVs are washed with PBS and subjected to another round of UC (100 000 g, 3 h). These steps are performed at a constant temperature of 4 °C. The extracted BEVs can be used immediately or stored at −80 °C for future applications. Figure was created with https://app.biorender.com/.

After detailing the biogenesis, types, and composition of BEVs, the focus shifts to their isolation, which is crucial for subsequent applications.^[12c]^ Various techniques, including ultracentrifugation (UC), ultrafiltration (UF), precipitation, affinity isolation, size exclusion chromatography, and density gradient centrifugation (DGC), have been developed to extract BEVs from fermentation broth.[^42^, ^50^] Although these methods generally achieve good yield and purity, complex fermentation media often necessitate combining techniques to eliminate contaminants.^[44a]^ This combining approach has been successfully applied to isolate BEVs from complex sources with high purity and consistency (**Figure** **4**).[^22^, ^51^] Following isolation, techniques such as transmission electron microscopy, nanoparticle tracking analysis, and western blot are used to assess the sizes, shapes, and concentrations of BEVs.^[41a]^

### 3.2 Natural BEVs‐Based Gut‐Bone Axis for OP Therapy

**Figure 5:**
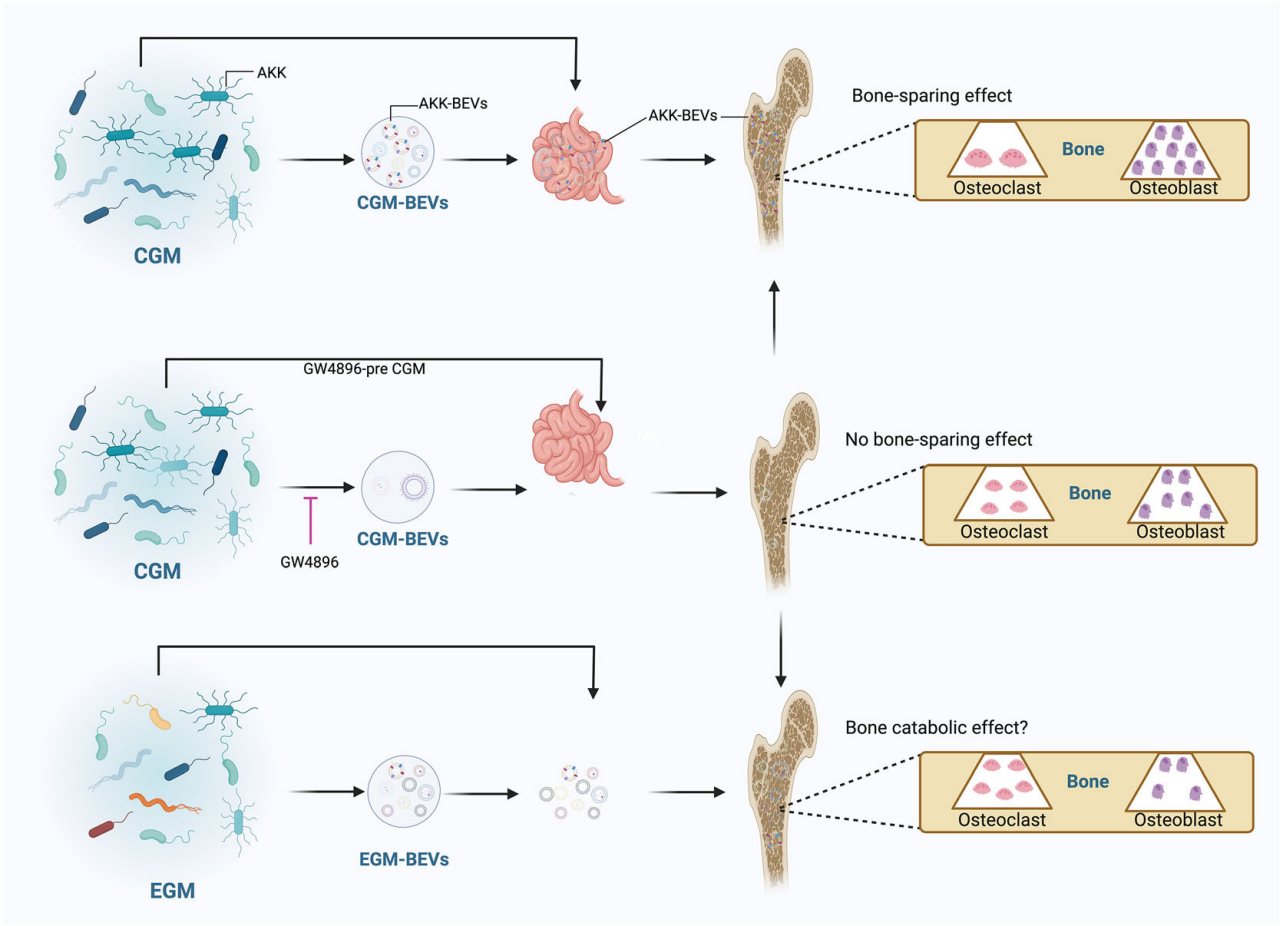
Natural BEVs‐based gut‐bone axis for OP therapy. Schematic diagram of CGM‐derived BEVs and AKK‐derived BEVs for OP therapy. Figure was recreated with https://app.biorender.com/. Reproduced with permission.^[22a]^ Copyright 2022, Elsevier Ltd.

GM, such as *Lactobacillus reuteri,* ^[^ ^52^ ^]^ LGG,[^6^, ^35^] and *Akkermansia muciniphila* (AKK)^[22a]^ have been reported to be inextricably linked to bone metabolism and OP. Recently, it has been acknowledged that the communications between bacteria and cells mainly rely on BEVs, which can transport a multitude of bioactive molecules to distant tissues or cells to regulate bone metabolism. Liu et al.^[22a]^ found that children microbiota colonization instead of older microbiota colonization improved OP in ovariectomy (OVX)‐induced osteoporotic mice. Subsequently, 16S rRNA gene sequencing showed that the children GM (CGM) had more abundant AKK compared with elderly GM. Importantly, the supplementation of AKK is sufficient to compensate for OVX‐induced OP. Both CGM and AKK‐derived BEVs could enter bone tissue to enhance osteogenic activity and inhibit osteoclasts, thereby alleviating OVX‐induced OP (**Figure** **5**). In addition, Wang et al.^[^ ^53^ ^]^ found that *Proteus mirabilis* (PM)‐derived BEVs could inhibit osteoclast formation. The mechanism proposes that PM‐BEVs robustly suppressed miR‐96‐5p expression, leading to increased Abca1 (ATP binding cassette subfamily A member 1) in osteoclasts and increased mitochondria‐dependent apoptosis. In conclusion, GM plays a crucial role in bone health by influencing OP through the transport of bioactive molecules via BEVs.

### 3.3 Engineered BEVs‐Based Gut‐Bone Axis for OP Therapy

#### 3.3.1 Engineering Modification Methods for BEVs

**Figure 6:**
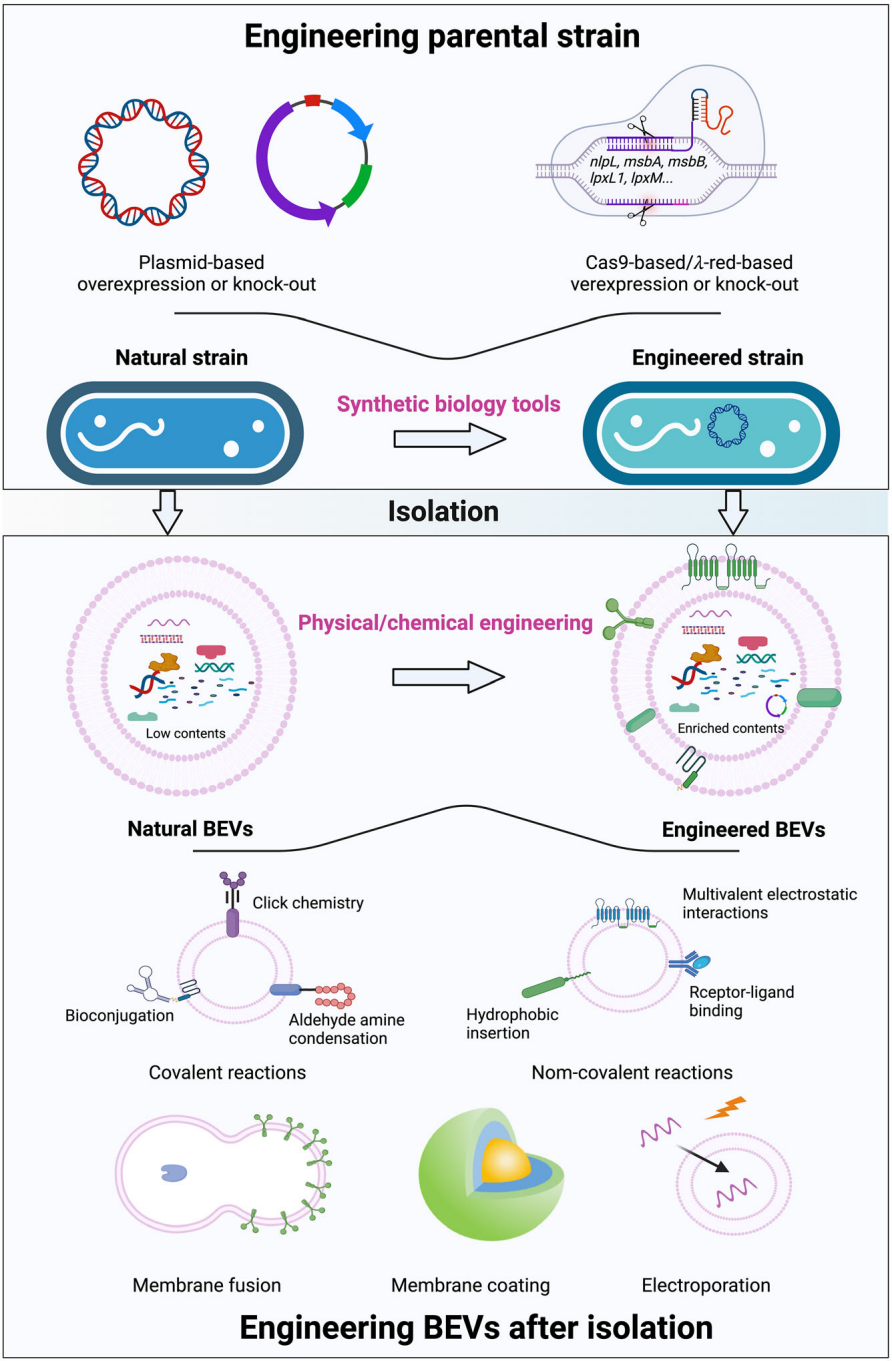
The strategies for modifying BEVs for OP treatment. The strategies include engineering the parental strain to produce therapeutic BEVs and modifying isolated BEVs. Naturally, BEVs come from natural strains, while engineered BEVs can originate from genetically modified strains or natural BEVs postisolation. Modifying the parental strain typically involves plasmid‐based or CRISPR‐Cas9‐based methods for overexpression or knockout. Similarly, altering isolated BEVs includes physical techniques like membrane fusion, membrane coating, and electroporation, as well as chemical methods such as click chemistry, aldehyde amine condensation, bioconjugation, hydrophobic insertion, multivalent electrostatic interactions, and receptor‐ligand binding reactions. Figure was created with https://app.biorender.com/.

Moreover, the therapeutic effect of these natural BEVs on bone degenerative diseases could be further improved through synthetic biology and physicochemical technologies.^[^ ^54^ ^]^ Therefore, we have comprehensively summarized engineering approaches, encompassing the modification of the parental strain and the engineering of BEVs after isolation (**Figure** **6**). Modifying parental strain mainly encompasses plasmid‐based strategy, such as CRISPR‐Cas9.^[^ ^55^ ^]^ Correspondingly, the modification of BEVs after isolation primarily involves physical engineering, such as membrane fusion and electroporation, as well as chemical engineering including covalent reactions and noncovalent reactions.^[^ ^56^ ^]^

#### 3.3.2 Engineered BEVs‐Based Gut‐Bone Axis for OP Therapy

**Figure 7:**
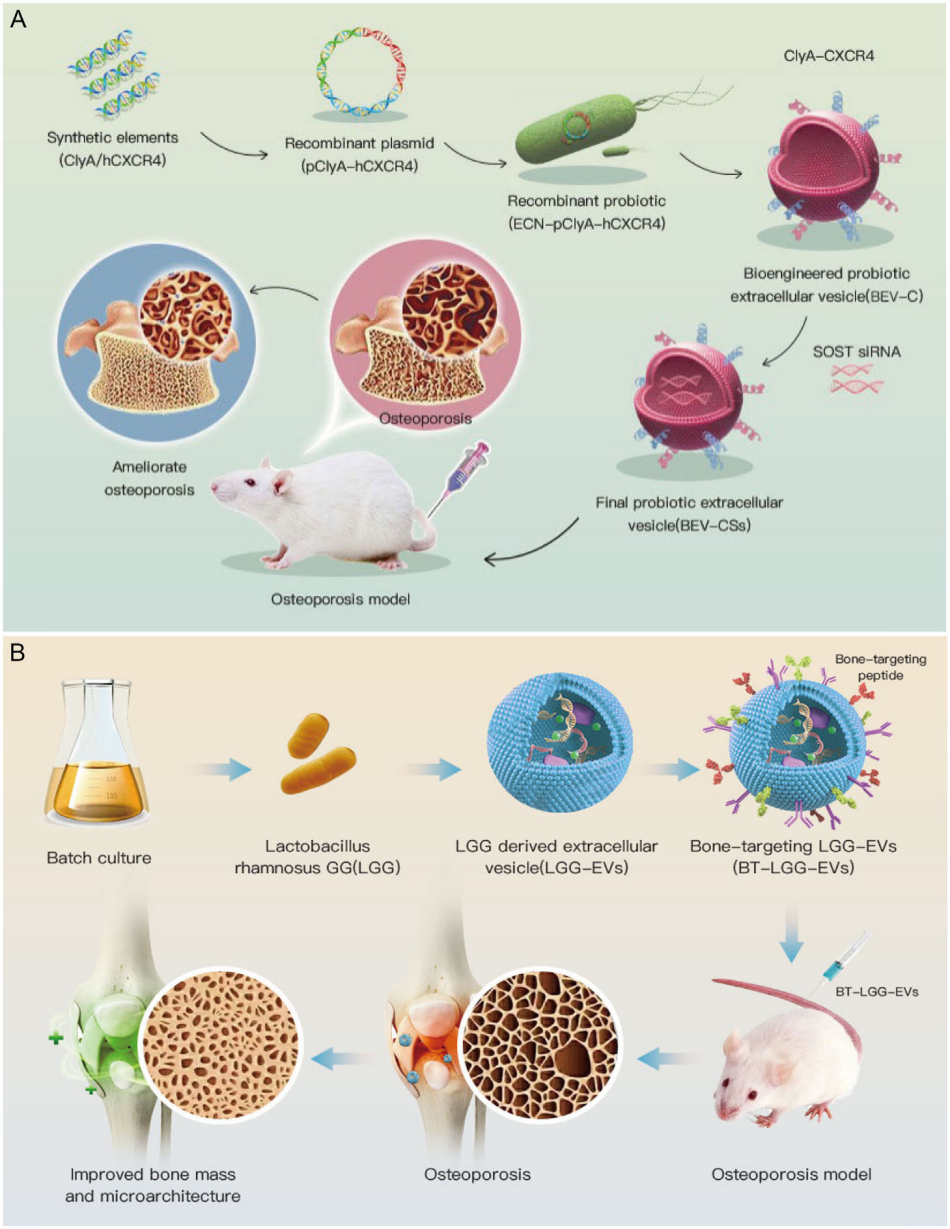
Engineered BEVs‐based gut‐bone axis for OP therapy. A) Schematic diagram of the construction of bone‐targeted BEVs for OP therapy. Reproduced with permission.^[^ ^4^ ^]^ Copyright 2023, Elsevier Ltd. B) Schematic diagram of preparation of bone‐targeted LGG‐EVs. Reproduced with permission.^[^ ^60^ ^]^ Copyright 2023, Elsevier Ltd.

The rapid advancement of synthetic biology gives BEVs more customization possibilities. Therefore, we first modified *Escherichia coli* Nissle 1917 to overexpress C‐X‐C motif chemokine receptor 4 (CXCR4^[^ ^57^ ^]^) on the membrane surface of BEVs by fusing BEVs surface protein ClyA.^[^ ^58^ ^]^ Furthermore, we used electroporation to load SOST siRNA into the BEVs, thereby constructing an engineered BEVs with bone targeting and bone treatment capabilities (**Figure** **7**A). Despite the rapid development of synthetic biology, Gram‐positive bacteria‐related genetic engineering methods still await further development. The thick cell wall and rich peptidoglycan layer of Gram‐positive bacteria LGG increase the difficulty of modifying the parent strain. Therefore, we used physical engineering methods to construct bone‐targeting BEVs. Diacyl lipid tail‐modified bone‐targeting peptides, SDSSD,^[^ ^59^ ^]^ were anchored on the LGG‐EVs membranes to generate bone‐targeted BEVs (BT‐LGG‐EVs), which could attenuate the OP by promoting osteogenic differentiation and inhibiting the formation of osteoclasts^[^ ^60^ ^]^ (Figure 7B).

Overall, both physicochemical‐based and synthetic biology‐based strategies were of vital significance for OP therapy. These studies demonstrated natural and engineered BEVs‐based gut‐bone axis regulation model, establishing a groundwork for the future applications of GM and presenting an innovative and promising therapeutic solution for OP treatment.

## 4 IOEVs‐Based Gut‐Bone Axis for OP Therapy

### 4.1 Intestinal Organoids for Bone Metabolism

**Figure 8:**
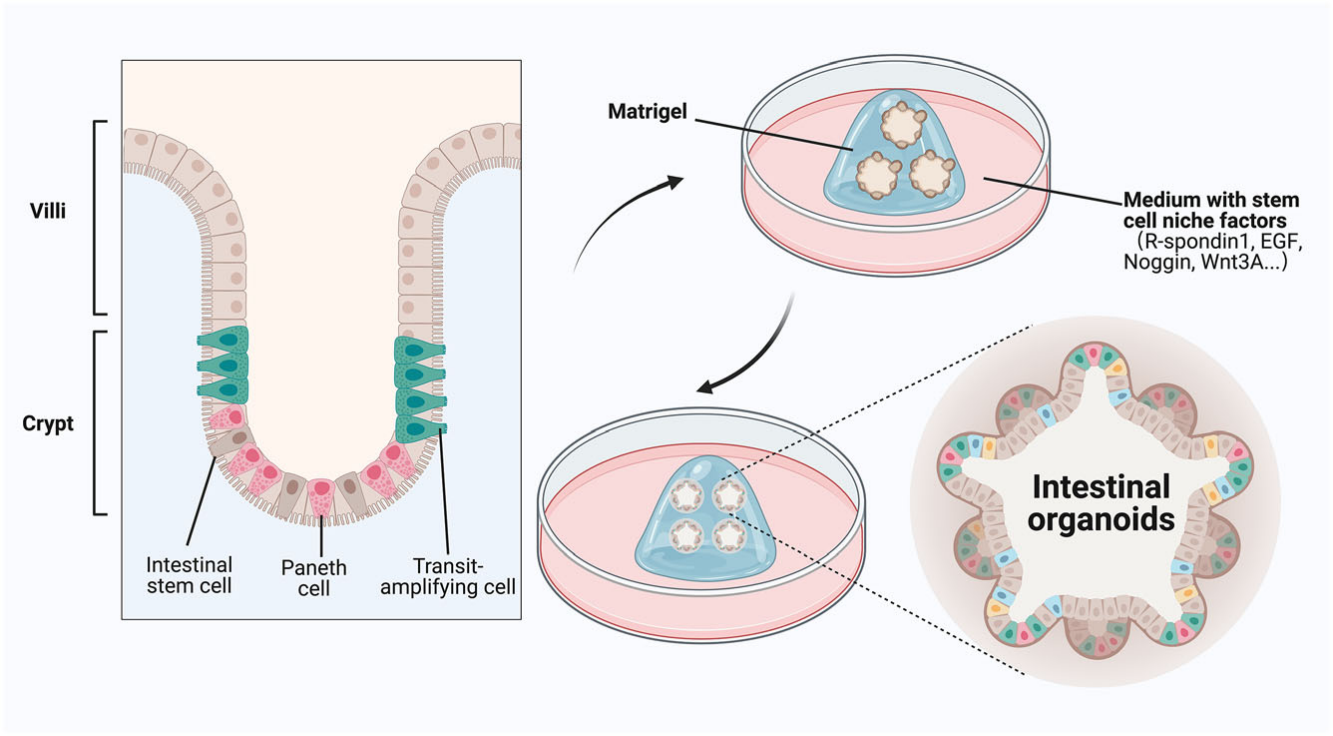
Schematic diagram of the construction of intestinal organoids. Reproduced with permission.^[^ ^20^ ^]^ Copyright 2023, Elsevier Ltd. Figure was created with https://app.biorender.com/.

Organoids, similar to real human tissues and organs to a certain extent, are 3D microorgans composed of a cluster of stem cells with the ability to self‐renew and self‐organize.[^14^, ^61^] Intestinal organoids are 3D cell clusters with crypt‐like buds that are self‐organized from intestinal stem cells in the presence of Matrigel and niche factors (such as R‐spondin1, EGF, Noggin, and Wnt3A) (**Figure** **8**). Currently, the most widely used applications of intestinal organoids are inflammatory bowel disease (IBD) chronic complication,^[^ ^62^ ^]^ mucosa injury and repair,^[^ ^63^ ^]^ intestinal microecology exploration,^[^ ^64^ ^]^ and IBD therapy.[^18^, ^65^] Recently, Watanabe et al.^[^ ^66^ ^]^ transplanted intestinal organoids into a mouse model of colitis, which laid a solid foundation for human clinical trials. Subsequently, their team cultured stem cells from the patient's healthy intestines to construct intestinal organoids for the treatment of IBD and achieved great therapeutic results. Although the treatment of IBD has achieved certain results, the pathogenesis of IBD has not been fully elucidated.^[^ ^67^ ^]^ It has been reported that the factors such as intestinal microbiota, drugs, diet, genetics, psychological state, and environment may contribute to the occurrence of IBD. Importantly, these factors also influence bone metabolism.^[^ ^68^ ^]^ Lower bone formation rates and fewer osteoblasts were observed in various intestinal inflammatory diseases.^[^ ^69^ ^]^ These results indicate a close connection between intestinal organoids and bone metabolism. Although there are currently few articles related to intestinal organoids and bone metabolism, there is no doubt that intestinal organoids and IOEVs have a bright future in regulating bone metabolism.

### 4.2 Overview of OEVs

#### 4.2.1 The Biogenesis, Types, and Composition of OEV

**Figure 9:**
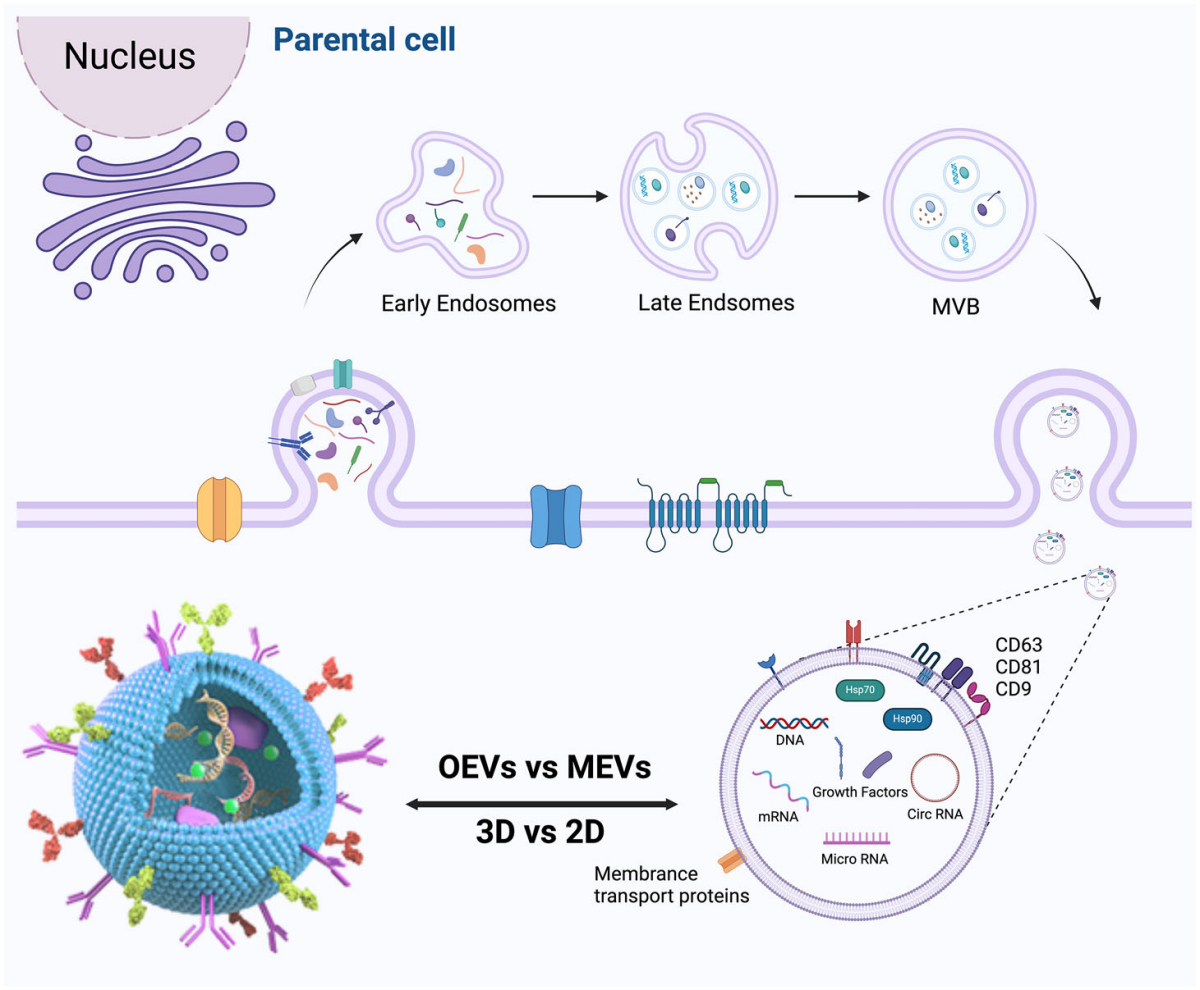
The biogenesis, structure, and composition of OEVs. The process initiates with the cytoplasmic membrane undergoing endocytosis to yield early endosomes. These evolve into multivesicular bodies harboring nascent vesicles within their endosomal interior through interactions with the Golgi apparatus, signifying the maturation of early endosomes into their late counterparts. Driven by specific proteins, these late endosomes can either merge with lysosomes for the breakdown of their contents or associate with the cytoplasmic membrane to extrude the intraluminal vesicles, effectively releasing OEVs. OEVs exhibit lipid bilayer structures containing a variety of proteins, nucleic acids, metabolites, and lipids. Figure was created with https://app.biorender.com/.

Mammalian cell‐derived EVs (MEVs), once considered mere carriers of cellular waste, have emerged as essential mediators of intercellular communication and are now recognized for their potential as next‐generation drug delivery platforms.^[^ ^70^ ^]^ The membrane surface of BEVs generally does not contain many specific proteins, while the membrane surface of MEVs contains a variety of specific proteins, such as tumor susceptibility gene 101 protein (TSG101), transmembrane proteins (CD9, CD63, and CD81), and apoptosis linked gene 2‐interacting protein X (Alix), which can be used as standards for subsequent characterization.^[^ ^71^ ^]^ In addition, MEVs also contain a variety of other proteins, nucleic acids, lipids, and other key components that regulate physiological and pathological processes.^[^ ^72^ ^]^ Since OEVs are isolated from mammalian cells after 3D culture, they are essentially a type of MEVs.[^19^, ^73^] Consequently, an in‐depth understanding of the biogenesis, structure, and composition of MEVs provides a comprehensive basis for understanding OEVs (**Figure** **9**).

#### 4.2.2 The Isolation of OEVs

**Figure 10:**
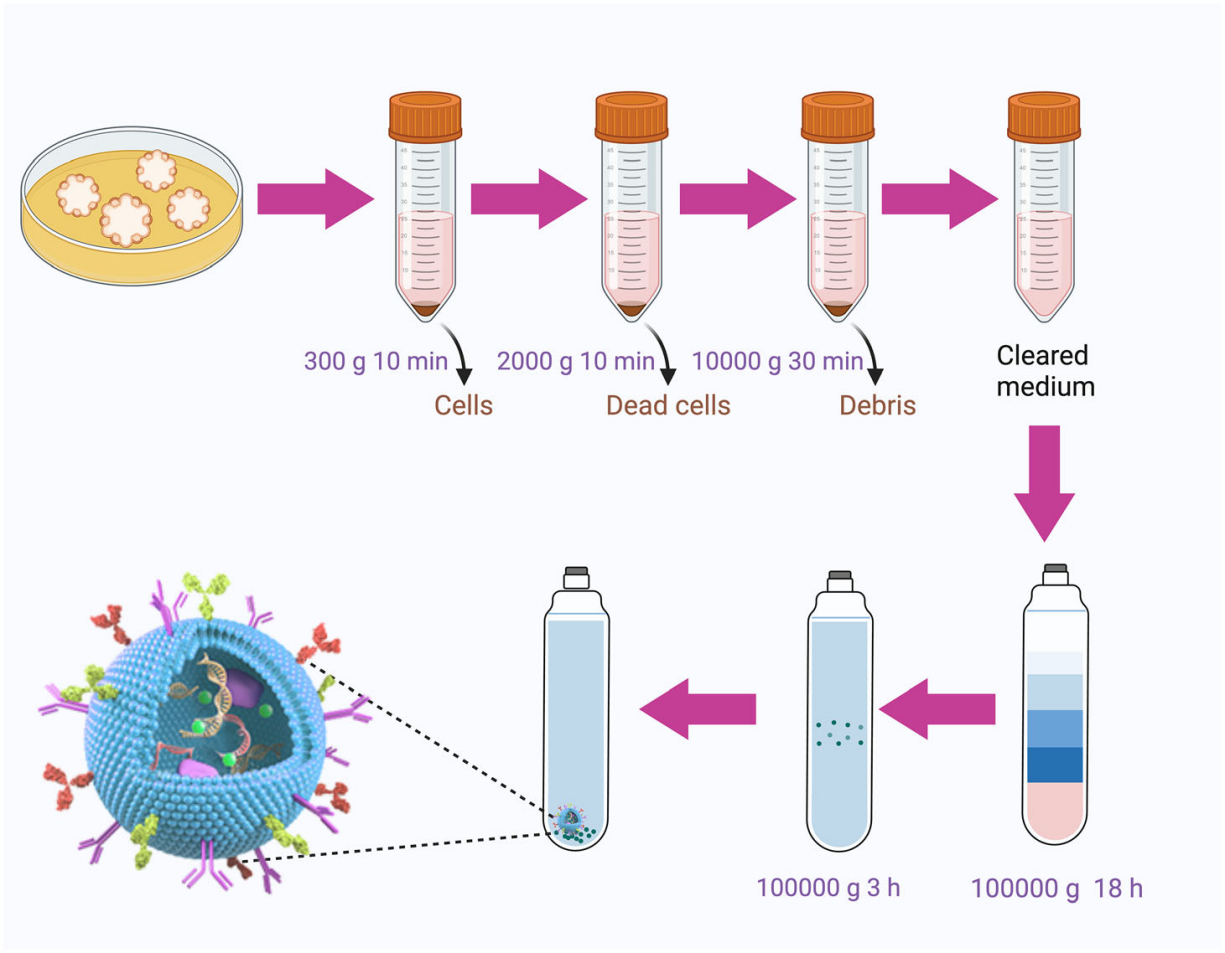
The isolation of OEVs. To isolate OEVs, the process typically begins with low‐speed centrifugation to remove matrix gel, intact cells, necrotic cells, and cellular debris. Subsequent UC at 100 000 g is used to gather OEVs, followed by a second round of UC at the same speed for purification. For further purification, it is recommended to employ DGC with iodixanol. The isolated OEVs are then resuspended in sterile phosphate‐buffered saline and stored at −80 °C for future use. Figure was created with https://app.biorender.com/.

The process of isolating MEVs through UC, including differential centrifugation, stands as the benchmark method and remains widely utilized and documented in the field.^[^ ^74^ ^]^ For OEVs isolation, initial steps typically involve low‐speed centrifugation (ranging from 300 to 10 000 g) to eliminate matrix gel, intact cells, necrotic cells, and cellular detritus (**Figure** **10**).^[^ ^75^ ^]^ Subsequently, UC at 100 000 g is deployed to collect OEVs. For purification of OEVs, a repeat UC at 100 000 g is conducted. In addition, for enhanced purification, DGC with iodixanol, is recommended. Finally, the collected OEVs were resuspended in sterile phosphate‐buffered saline and then stored at −80 °C for future use.

### 4.3 The Differences Between OEVs and MEVs

In contrast to conventional 2D cell culture systems, 3D cell cultures are capable of establishing tissue structures that closely mimic the environment of stem cell niches, presenting conditions more parallel to those found in human physiology.[^19^, ^76^] It has been discovered that although the production mechanisms and contents of the EVs were similar in MEVs and OEVs, the OEVs from the 3D culture exhibited superior functionality.^[^ ^77^ ^]^ Specifically, when OEVs were introduced to retinal photoreceptor neurons in coculture experiments, they induced higher levels of signaling factor secretion than MEVs. Further experiments involved administering these EVs to mice subjected to brain trauma, revealing that those treated with OEVs experienced markedly enhanced angiogenesis and neural regeneration compared to those receiving MEVs. Consequently, OEVs, with their greater abundance and enhanced biological effects, emerge as more advantageous for the treatment of related disorders than MEVs.

### 4.4 The Potential Role of IOEVs‐Based Gut‐Bone Axis for OP Therapy

**Figure 11:**
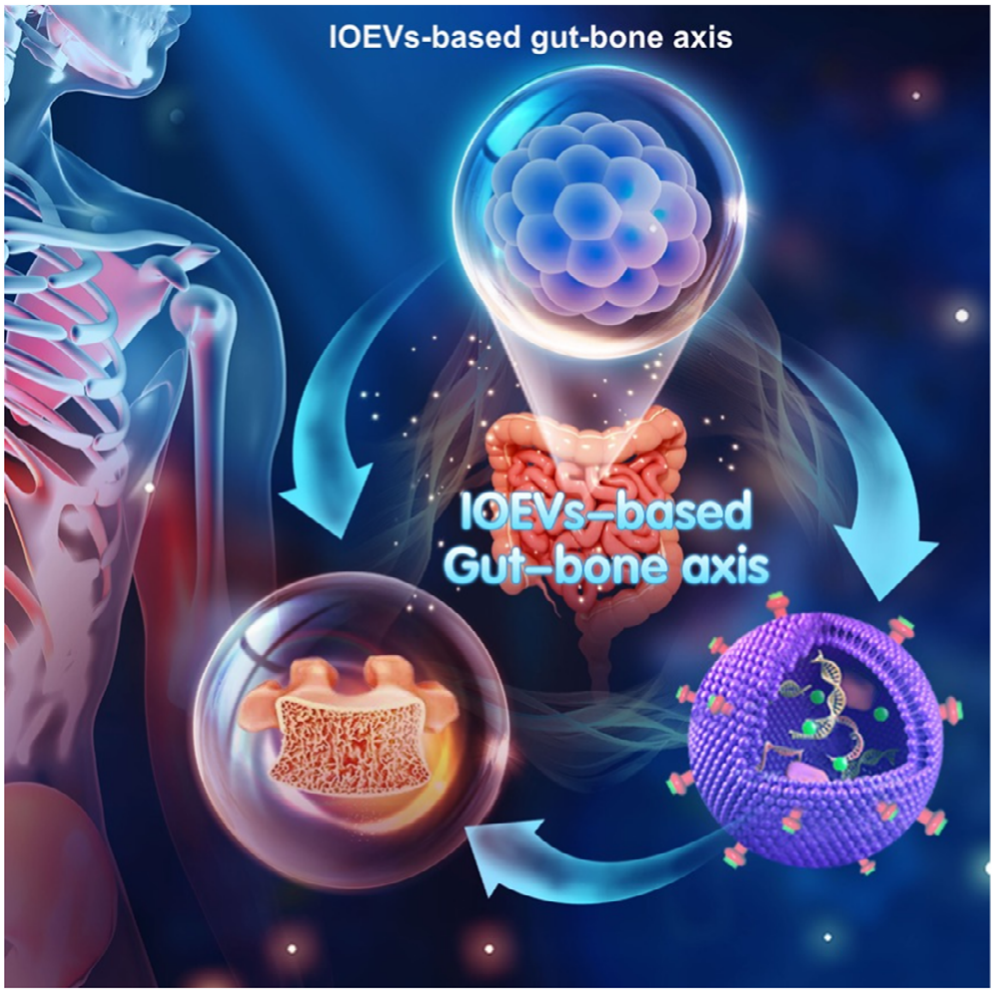
Schematic illustration of IOEVs‐based gut‐bone axis. The IOEVs‐based gut‐bone axis is a promising research direction that will provide new therapeutic strategy for the treatment of OP.

IOEVs, secreted by intestinal organoids, can regulate cell‐to‐cell communication by delivering endogenous nucleic acids, proteins, and lipids.^[^ ^78^, ^79^ ^]^ Zhang et al. found that IOEVs derived from mouse and human could regulate inflammatory responses of immune cells in vitro.^[^ ^78^ ^]^ IOEVs could also reduce endotoxin‐induced systemic inflammation and alleviate symptoms of DSS‐induced IBD in vivo. Subsequent mechanistic analysis revealed that multiple microRNAs, especially Let‐7, contributed to IOEV‐mediated immune regulation. In addition, OEVs are more beneficial for IBD treatment due to their more significant quantity and physiological effects than traditional EVs.^[17d]^ Therefore, we have innovatively proposed that intestinal organoids and IOEVs could be used for IBD treatment.^[^ ^20^ ^]^ Both intestinal organoids and IOEVs significantly improved IBD symptoms and improved bone mass. Therefore, we propose a brand‐new perspective that IOEVs‐based gut‐bone axis may provide novel insight into the treatment of OP (**Figure** **11**).

**Figure 12:**
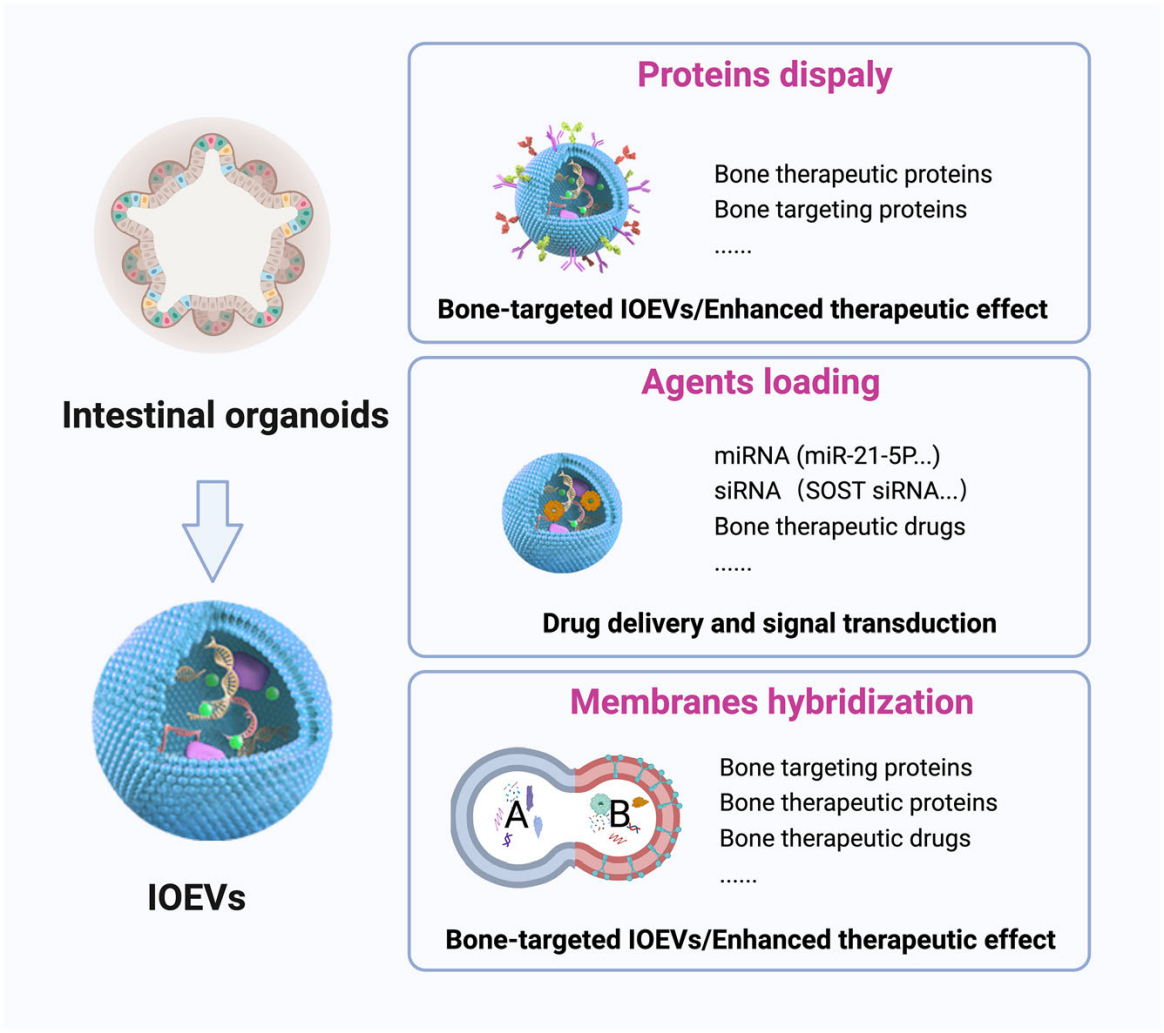
The potential role of IOEVs‐based gut‐bone axis for OP therapy. Displaying bone‐targeting proteins or bone therapeutic proteins on the membranes, loading bone therapeutic miRNA, siRNA, and drug, and hybridizing other functionalized biological membranes can be used to enrich the therapeutic and targeting functions of IOEVs for OP therapy. In conclusion, IOEVs‐based gut‐bone axis will provide a promising insight into the treatment of OP. Figure was created with https://app.biorender.com/.

Notably, the development of engineering methods, including modifying parental cells to create engineering IOEVs and modifying IOEVs after isolation, has also endowed IOEVs with customizability, making IOEVs a strong candidate for clinical application in orthopedics (**Figure** **12**). With the application of synthetic biology in the construction of organoids,^[14c]^ synthetic biology methods will be used in the future to transform parental cells to generate IOEVs with bone treatment and bone‐targeting capabilities. Moreover, either modifying the parent cells to create engineered IOEVs or modifying the IOEVs after isolation can be used to confer extra‐membranous and intra‐membranous bone therapeutic and bone‐targeting capabilities to IOEVs. For example, the bone‐targeting protein (such as CXCR4) and bone therapeutic protein (such as BMP‐2) can be displayed on the extra‐membranous. The miRNAs (such as miR‐21‐5p, miR‐25‐3p, let‐7b‐5p) with bone therapeutic functions, the siRNA (such as SOST siRNA), and drugs (such as PTH, alendronate) can be loaded in the intra‐membranous. These will enhance the therapeutic ability of IOEVs for OP and better reflect the significance of IOEVs‐based gut‐bone axis.

## 5 Main Challenges and Possible Solutions of EVs‐Based Gut‐Bone Axis

As mentioned above, although the therapeutic applications of BEV‐based and IOEV‐based gut‐bone axis bring great promise for OP treatment, there are still several obstacles to transferring them from the laboratory to the clinic. Here, we summarize the main challenges and possible solutions for BEVs‐based and IOEVs‐based gut‐bone axes. Major challenges include safety and biocompatibility, standardization and quality control, and scalable production and cost‐effectiveness ratio.

### 5.1 Safety and Biocompatibility

The foremost consideration for any new therapy transitioning to clinical application is its safety and biocompatibility. BEVs‐based OP treatments have received safety certification in animal experiment.^[22a]^ Intestinal organoids and IOEVs‐based treatments have even yielded one clinical trial result.^[^ ^66^ ^]^ Although the regulatory approval process for new therapies is often complex and time‐consuming, as relatively new therapies, BEVs and IOEVs still need to undergo extensive preclinical trials to demonstrate their safety and tolerability in humans, and to prove their advantages over existing treatments.

### 5.2 Standardization and Quality Control

The process of extracting, purifying, and modifying BEVs and OEVs requires high standardization to ensure consistency and reproducibility across batches for clinical applications.^[^ ^80^ ^]^ The variability introduced by different isolation techniques can result in significant heterogeneity among BEVs and OEVs, undermining the consistency and reliability of research findings.^[^ ^81^ ^]^ Even different storage conditions can affect the heterogeneity of BEVs and OEVs.^[^ ^82^ ^]^ Currently, a lack of unified production and quality control standards poses a barrier to their clinical application. Addressing these issues through a better understanding of BEVs and OEVs heterogeneity and the establishment of standardized production and isolation practices. For example, strict implementation of the Minimal Information for Studies of Extracellular Vesicles (such as MISEV2018, MISEV2023) guidelines issued by the International Society for Extracellular Vesicles (ISEV) will also significantly advance standardization in this field.[^11^, ^83^]

### 5.3 Scalable Production and Cost‐Effectiveness Ratio

While BEVs and IOEVs can be successfully obtained and engineered at the laboratory scale, challenges remain in utilizing cost‐effective methods to scale up their production to meet clinical needs.^[^ ^84^ ^]^ The process of developing new therapies is costly. Ensuring that BEVs‐based and IOEVs‐based strategies are both economical and effective is an important consideration. The cost of treatment needs to be within an acceptable range to promote widespread adoption. Bioreactor fermentation is a cost‐effective and scalable biotechnological method that facilitates the rapid cultivation of cells, both prokaryotic and eukaryotic, and subsequent harvesting of large quantities of their metabolic products, such as BEVs and OEVs.^[^ ^85^ ^]^

## 6 Conclusion and Perspective

**Figure 13:**
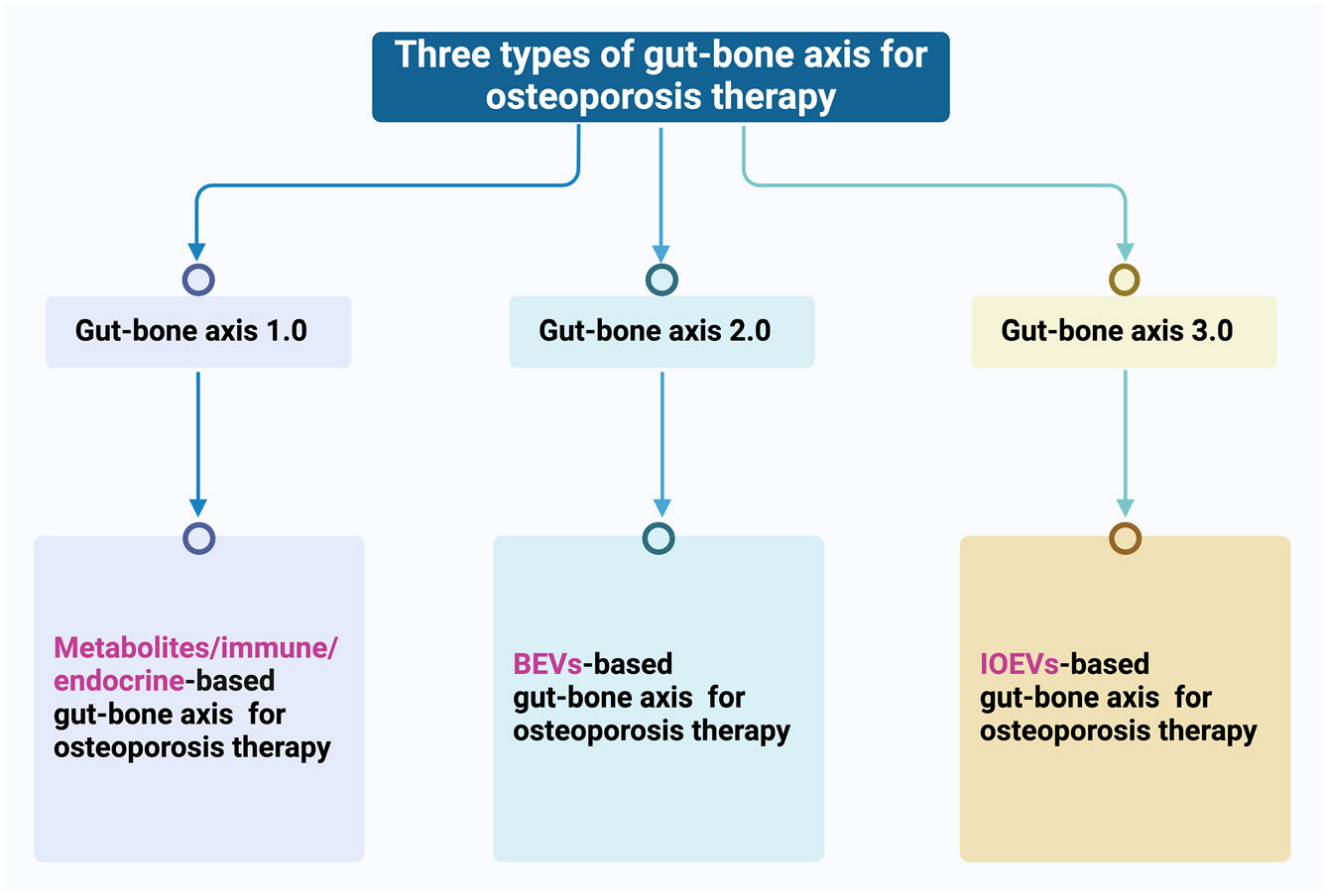
Three types of gut‐bone axes for OP therapy. Within the three types of the gut‐bone axes, the conventional gut‐bone axis is a more intricate and comprehensive model, where BEVs and IOEVs emerge as novel mediators influencing the physiological interactions between the gut and bone. Figure was created with https://app.biorender.com/.

OP is a complex metabolic bone disease, the molecular mechanism of which involves the functional imbalance of osteoblasts and osteoclasts, the regulation of hormones and cytokines, the influence of genes, and the role of microorganisms. Procollagen type I N‐terminal peptide (PINP) and C‐terminal collagen peptide (CTX) are two important biomarkers that are commonly used to assess the state of bone metabolism, especially in the diagnosis and monitoring of OP. Here, we provided a comprehensive summary of traditional gut‐bone axis, BEVs‐based gut‐bone axis, and IOEVs‐based gut‐bone axis for OP therapy (**Figure** **13**). Traditional gut‐bone axis includes metabolites‐based (directly) and immune and endocrine systems‐based (indirectly). Then, we mainly summarize the BEVs‐based and IOEVs‐based gut‐bone axes for OP therapy. BEVs, a phospholipid bilayer secreted by bacteria, could regulate bone metabolism by delivering endogenous bioactive agents, such as nucleic acids, proteins, and lipids. The applications of natural and engineered BEVs, including Gram‐positive BEVs and Gram‐negative BEVs, in the treatment of OP in recent years have been summarized. However, traditional gut‐bone axis and BEVs‐based gut‐bone axis may not fully reveal the real relationship between gut and bone. Therefore, we innovatively proposed the concept of IOEVs‐based gut‐bone axis, where IOEVs may also regulate bone metabolism by delivering endogenous nucleic acids, proteins, and lipids.

In the three types of the gut‐bone axes, the traditional gut‐bone axis is expanded into a more comprehensive and complex model. BEVs and IOEVs, as new communication media between the gut and bone, introduce more physiological influencing factors. Studies have shown that the GM can influence bone health through the production of metabolites.^[^ ^86^ ^]^ By analyzing specific metabolites produced by the GM, new biomarkers may be developed for early detection and assessment of OP risk. This can be achieved through metabolite detection in stool samples or blood. In addition, by adjusting the diet and using probiotics or prebiotics, the composition and function of the intestinal microbiota can be influenced, thereby having a positive impact on bone metabolism. It has been reported that GM can affect osteoclasts, osteoblasts, and other cell types within the basic multicellular unit (BMU) by secreting BEVs, thereby regulating bone homeostasis. BEVs are important carriers of intercellular communication and can carry molecules such as proteins, mRNA, miRNA, etc., which affect the bone formation and remodeling processes.^[^ ^87^ ^]^ Overall, BEVs regulate BMU activity through interaction with these cell types, thus exerting a significant impact on bone homeostasis and overall bone health, providing an important basis for the development of novel therapeutic approaches using BEVs in the gut‐bone axis. By studying the vital EVs in the BEVs‐based or IOEVs‐based gut‐bone axis, new treatments may be developed, such as the use of engineered BEVs or IOEVs to enhance bone formation or reduce bone resorption. The purpose of this concept is to explore the combined effects of intestinal microbes, BEVs, and intestinal organoids on bone health. By studying the intrinsic connections between the three types of the gut‐bone axes, we can gain a deeper understanding of the complex interactions between the gut and bone, providing guidance for the development of more effective treatments and interventions. This comprehensive research model helps reveal the interaction between the gut and bone, providing new perspectives and opportunities for the treatment and prevention of OP.

Metabolites derived from bacteria are estimated to account for ≈10% of circulating metabolites.^[^ ^88^ ^]^ The rapid advances in metabolomics will lead to the discovery of metabolites critical to the regulation of bone metabolism as well as possible new immune‐metabolic pathways, which will provide innovative therapeutic opportunities for OP therapy. In addition, both probiotics and pathogenic bacteria have been reported to be able to improve OP through BEVs.^[^ ^53^ ^]^ Indeed, BEVs have the capability to traverse the intestinal epithelial barrier and initiate immune regulation through interactions with immune cells located in the lamina propria.^[^ ^89^ ^]^ Therefore, the BEVs‐based oral bacterial strategies to regulate immune cells (such as Treg) may be a promising direction for OP treatment. In addition, synthetic biology has given infinite possibilities to traditional gut‐bone axis and BEVs‐based gut‐bone axis.^[^ ^90^ ^]^ Bacteria possess the benefits of swift proliferation, extensive gene editing techniques, and well‐established high‐density cultivation methods.[^85^, ^90^] Therefore, we are able to customize various engineered bacteria and engineered BEVs for OP therapy.

At present, MEVs,^[^ ^91^ ^]^ plant‐derived EVs,^[^ ^92^ ^]^ and BEVs^[^ ^93^ ^]^ have formed three‐legged situation. Since OEVs have higher yield and closer in vivo metabolism than that of traditional MEVs,^[17d]^ it will become an important and integral part of MEVs. In addition, with the continued boom in organoid research, OEVs will also receive more attention.^[94a,b]^ Recently, Trentesaux et al.^[14c]^ harnessed synthetic biology to engineer multifunctional organoids. Our team also proposed the concept of artificial intelligence (AI)‐enabled organoids.^[^ ^95^ ^]^ AI interfacing enables efficient intestinal organoid construction, multiscale image analysis, and precise preclinical evaluation, including rapid screening to optimize experimental designs and cost‐effective extraction of image features for better understanding. It emphasizes the importance of efficient multiomics analysis and accurate preclinical evaluation to gauge AI's performance in clinical settings. Last, it highlights the need to find effective ways to apply these theories in practice to fully leverage AI in the development of AI‐enabled organoids. With the introduction of AI^[^ ^96^ ^]^ and synthetic biology,^[14c]^ we are able to customize a variety of powerful IOEVs for the treatment of complex OP.^[^ ^97^ ^]^

Moreover, three types of gut‐bone axes will also be a powerful tool for OPF, the most serious complication of OP. The combination of three types gut‐bone axes and various composite materials, such as hydrogels^[^ ^98^ ^]^ and scaffolds,^[^ ^99^ ^]^ can be better used for OPF repair. Despite ongoing challenges, unremitting research on the gut‐bone axis will undoubtedly generate innovative and efficient solutions for OP and its complications.

## Abbreviations

**Table:** 

Activating transcription factor 4	ATF4
*Akkermansia muciniphila*	AKK
Artificial intelligence	AI
Bacterial EVs	BEVs
Bone‐targeted BEVs	BT‐LGG‐EVs
Children GM	CGM
C‐X‐C motif chemokine receptor 4	CXCR4
Extracellular vesicles	EVs
Gut microbiota	GM
Histone deacetylases	HDACs
Human Microbiome Project	HMP
Hydrogen sulfide	H_2_S
Inflammatory bowel disease	IBD
insulin‐like growth factor 1	IGF‐1
Integrative Human Microbiome Project	iHMP
Intestinal OEVs	IOEVs
*Lactobacillus rhamnosus* GG	LGG
mammalian EVs	MEVs
Organoids EVs	OEVs
Osteoporosis	OP
Osteoporosis fracture	OPF
Ovariectomy	OVX
Parathyroid hormone	PTH
Parathyroid hormone	PTH
Plant‐derived EVs	PEVs
Procollagen type I N‐terminal peptide	PINP, C‐terminal collagen peptide (CTX)
*Proteus mirabilis*	PM
Receptor activator of nuclear factor kappa‐B ligand	RANKL
Runt‐related transcription factor 2	RUNX2
Short‐chain fatty acids	SCFAs
Sp7 transcription factor	Sp7, Osterix
T helper 17 cells	Th17
T Regulatory	Treg
Three‐dimensional	3D

## Conflict of Interest

The authors declare no conflict of interest.

## Author Contributions

**Han Liu**: conceptualization: (lead); funding acquisition: (lead); visualization: (lead); writing—original draft: (lead); writing—review & editing: (lead). **Ruiyang Li**: conceptualization: (supporting); writing—original draft: (supporting); writing—review & editing: (supporting). **Huijian Yang**: writing—original draft: (supporting); writing—review & editing: (supporting). **Bo Situ**: conceptualization: (equal); formal analysis: (equal); investigation: (equal); writing—original draft: (equal); writing—review & editing: (equal). **Guangchao Wang**: conceptualization: (equal); supervision: (equal); writing—review & editing: (equal). **Ke Xu**: conceptualization: (equal); visualization: (equal); writing—original draft: (equal); writing—review & editing: (equal). **Jiacan Su**: visualization: (equal); writing—original draft: (equal); writing—review & editing: (equal).
